# The minimal SUF system is not required for Fe–S cluster biogenesis in the methanogenic archaeon *Methanosarcina acetivorans*

**DOI:** 10.1038/s41598-023-42400-x

**Published:** 2023-09-13

**Authors:** Jasleen Saini, Thomas M. Deere, Daniel J. Lessner

**Affiliations:** Department of Biological Sciences, University of Arkansas-Fayetteville, Fayetteville, AR USA

**Keywords:** Microbiology, Archaea, Microbial genetics, Biochemistry, Metals

## Abstract

Iron–sulfur (Fe–S) proteins are essential for the ability of methanogens to carry out methanogenesis and biological nitrogen fixation (diazotrophy). Nonetheless, the factors involved in Fe–S cluster biogenesis in methanogens remain largely unknown. The minimal SUF Fe–S cluster biogenesis system (i.e., SufBC) is postulated to serve as the primary system in methanogens. Here, the role of SufBC in *Methanosarcina acetivorans*, which contains two *sufCB* gene clusters, was investigated. The CRISPRi-dCas9 and CRISPR-Cas9 systems were utilized to repress or delete *sufC1B1* and *sufC2B2*, respectively. Neither the dual repression of *sufC1B1* and *sufC2B2* nor the deletion of both *sufC1B1* and *sufC2B2* affected the growth of *M. acetivorans* under any conditions tested, including diazotrophy. Interestingly, deletion of only *sufC1B1* led to a delayed-growth phenotype under all growth conditions, suggesting that the deletion of *sufC2B2* acts as a suppressor mutation in the absence of *sufC1B1*. In addition, the deletion of *sufC1B1* and/or *sufC2B2* did not affect the total Fe–S cluster content in *M. acetivorans* cells. Overall, these results reveal that the minimal SUF system is not required for Fe–S cluster biogenesis in *M. acetivorans* and challenge the universal role of SufBC in Fe–S cluster biogenesis in methanogens.

## Introduction

Iron–sulfur (Fe–S) clusters are ancient inorganic cofactors present in cells from all domains of life, where they serve numerous biological functions. Notably, Fe–S clusters serve a critical role in electron transfer in various metabolic enzymes, with [2Fe–2S], [3Fe–4S], and [4Fe–4S] clusters being the most common^1^. Decades of research have demonstrated that the formation of Fe–S clusters within cells is not spontaneous but requires dedicated protein machinery to synthesize and transfer clusters to apo-proteins. The NIF (nitrogen fixation) system dedicated to the biogenesis of the simple and complex Fe–S clusters in the components of nitrogenase was the first Fe–S cluster biogenesis system discovered^2^. Nitrogenase is required for biological nitrogen fixation (diazotrophy) by prokaryotes^3^. Subsequently, the general Fe–S cluster biogenesis systems were discovered in bacteria—ISC (iron–sulfur cluster) and SUF (sulfur formation) systems. *Escherichia coli* contains both ISC and SUF, with ISC serving as the housekeeping system and SUF being important during stress conditions^4^. However, some bacteria only contain SUF (e.gs., *Bacillus subtilis* and *Mycobacterium tuberculosis*) where it serves as the primary system^5,6^. Eukaryotes contain homologous ISC and SUF systems housed within Fe–S protein containing organelles, with ISC found in mitochondria and SUF within plastids. An additional system, CIA (cytosolic iron sulfur assembly) supports the biogenesis of cytosolic and nuclear Fe–S cluster proteins in eukaryotes^7^. The minimal components of the NIF, ISC, and SUF systems are a cysteine desulfurase (IscS, SufS, and/or NifS) that liberates sulfur from L-cysteine and transfers it to a scaffold protein (IscU, SufBC, and/or NifU) where the Fe–S cluster is assembled. The Fe–S cluster is then transferred directly to a target apo-protein or to an intermediate carrier protein^4^.

Aerobic organisms have developed metabolic pathways that rely less on Fe–S proteins due to the vulnerability of Fe–S clusters to oxygen. Oxygen can cause the disassembly of Fe–S clusters and generate highly toxic hydroxyl radicals^8^. As such, the genomes of obligate anaerobes encode substantially more Fe–S proteins than obligate and facultative aerobes^9^. In particular, anaerobic acetogenic bacteria (acetogens) and anaerobic methanogenic archaea (methanogens) are predicted to contain the highest percentage of Fe–S proteins per genome^10^. For example, the number of Fe–S proteins in the methanogen *Methanococcus maripaludis* is ~ 15-fold higher than facultative *E. coli*^11^. Methanogens contain the largest number of Fe–S cluster proteins among archaea and Fe–S proteins are critical to their ability to produce methane and fix dinitrogen. The methanogenic CO_2_ reducing enzyme formyl-methanofuran dehydrogenase (Fwd) and heterodisulfide reductase (Hdr-Mvh) involved in the first and last steps of methanogenesis respectively, contain multiple Fe–S clusters^12,13^. Nitrogenase contains a simple [4Fe-4S] cluster and complex [8Fe-7S] and [7Fe-9S-Mo-C-homocitrate] clusters, referred to as the P- and M-clusters, respectively^3,14^.

Components of the SUF system are present in diverse species across all domains of life. The *suf* operon contains six genes in *E. coli*—*sufA*, *sufB*, *sufC*, *sufD*, *sufS*, and *sufE*. SufE acts as a sulfur acceptor from the cysteine desulfurase SufS, thereby enhancing the activity of SufS. SufSE donates sulfur to the scaffold formed by the SufBC_2_D complex. SufA is suggested to be a Fe–S cluster carrier protein that delivers Fe–S clusters to the target apo-proteins^15^. SufB_2_C_2_ can also function as an Fe–S cluster scaffold^16^. Virtually all archaea encode homologs of the core components of the SUF system (SufBC) shown to function in Fe–S cluster biosynthesis in bacteria and eukaryotes^17^. Recently, this minimal Fe–S cluster biogenesis system comprising only of the SUF scaffold proteins (SufB and SufC) was suggested to be renamed to SMS (SUF-like minimal system). The minimal SUF system (SMS) is common in archaea, while the complex SUF system (SufABCDSU) is common in bacteria^18^. Nearly all methanogens encode SufBC and some encode IscSU, the minimal components of the ISC system. Methanogens do not encode components of the NIF system despite many methanogens containing nitrogenase and being capable of fixing dinitrogen^17,19^. *M. maripaludis* contains only SufBC but no cysteine desulfurase (neither IscS nor SufS). Importantly, unlike other characterized organisms, *M. maripaludis* was shown to use sulfide as the sulfur donor to Fe–S clusters and does not use cysteine as a sulfur source^11^. Hence, sulfide-dependent Fe–S cluster assembly in *M. maripaludis* likely involves SufBC and it is hypothesized that SufBC serves as the core Fe–S cluster biogenesis system in methanogens. Results from a transposon mutagenesis screening indicated that SufBC is essential to *M. maripaludis*^20^. Thus, bioinformatics analysis and some experimental evidence support that SufBC is essential to Fe–S cluster biogenesis in methanogens^17,18, 21^.

*Methanosarcina acetivorans* contains two copies of SufBC (SufBC1-2) and three copies of IscSU (IscSU1-3). *M. acetivorans* can also use either sulfide or cysteine as a sulfur source. IscS2U2 was recently shown to function in Fe–S cluster biogenesis and deletion of *iscS2U2* slightly impaired the growth of *M. acetivorans* when cysteine was the sulfur source^19^. Thus, it was hypothesized that IscSU may function in cysteine-specific Fe–S cluster biogenesis, whereas SufBC may function in sulfide-specific Fe–S cluster biogenesis in *M. acetivorans*^19^. More recently, recombinant *M. acetivorans* SufB2C2 was shown to be capable of in vitro Fe–S cluster transfer consistent with *M. acetivorans* containing a functional minimal SUF Fe–S cluster biogenesis system^18^. *M. acetivorans* also contains all three types of nitrogenase and is capable of Mo-dependent and Mo-independent diazotrophy^22,23^. However, *M. acetivorans* lacks NifSU, components of the NIF system, specific to nitrogenase Fe–S cluster biogenesis in most bacteria. It was recently suggested that SufBC in methanogens, including *M. acetivorans*, is required for Fe–S cluster assembly in nitrogenase^18^. However, the in vivo importance of the minimal SUF system (or SMS) to *M. acetivorans*, and methanogens in general, is largely unknown. To assess the in vivo importance of SufB1C1 and SufB2C2 to *M. acetivorans*, we used the recently developed CRISPRi-dCas9 (CRISPRi) repression and CRISPR-Cas9 gene editing systems to repress and delete *sufC1B1* and *sufC2B2*, respectively^22,24^. The impact of repression and deletion of *sufCB* on the growth of *M. acetivorans* under several conditions was tested.

## Results

### SufB2 and SufC2 form a complex within *M. acetivorans*

**Figure 1:**
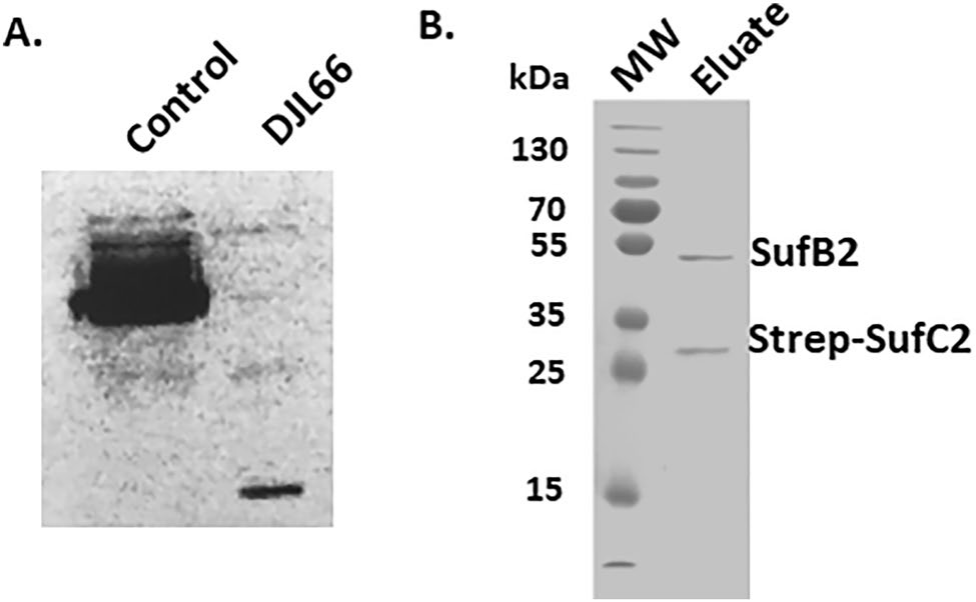
Purified strep-SufC2 forms a complex with SufB2. (**A**) Detection of strep-SufC2 expressed in strain DJL66 by Western blot using strep-specific antibodies. Lysate from strain WWM73 (pSK2) that expresses strep-UidA was used as a control. The uncropped image of the Western blot is shown in Fig. S1. (**B**) SDS-PAGE analysis of eluate from the purification of strep-SufC2.

Recombinant *M. acetivorans* SufB2 and SufC2 expressed in *E. coli* were previously shown to form a complex capable of Fe–S cluster assembly and transfer^18^. The ability of SufB2 and SufC2 to form a stable heterocomplex in *M. acetivorans* was assessed by first generating *M. acetivorans* strain DJL66 (Table 1), that in addition to containing *sufC2B2*, contains an integrated plasmid expressing strep-tagged SufC2 (strep-SufC2) from a second *sufC2B2* gene cluster. Expression of strep-SufC2 in *M. acetivorans* strain DJL66 was confirmed by Western blot with strep-tag specific antibodies (Fig. 1A). Strain DJL66 grew comparably to parent strain WWM73 under all growth conditions tested (data not shown), indicating expressed strep-SufC2 does not impact the physiology of *M. acetivorans*. Strep-SufC2 was purified from strain DJL66 cells using streptavidin resin and elution with desthiobiotin. Eluate was analyzed by SDS-PAGE and two distinct protein bands were observed consistent with the MW of strep-SufC2 and SufB2 (Fig. 1B). Each band was excised, trypsin-digested, and unique peptides specific to SufC2 and SufB2 were identified by mass spectrometry (Fig. S2), confirming the purification of SufB2 along with strep-SufC2. The purified strep-SufC2B2 complex was colorless, and the UV–visible spectrum was consistent with the absence of Fe–S clusters (data not shown). Overall, these results demonstrate that SufB2 and SufC2 form a stable heterocomplex in *M. acetivorans* consistent with previous results with recombinant protein expressed in *E. coli*^18^.

**Table 1:** *M. acetivorans* strains used in this study.

Strain	Genotype	Relevant properties	Reference
WWM73	Δhpt::PmcrB-tetR-C31-int-attP	Pseudo-wild type parent strain	^25^
DJL66	WWM73 att::pDL365	Produces strep-SufC2	This study
DJL72	WWM73 att::pDL734	gRNA-free control strain	^22^
DJL130	WWM73 att::pDL366	CRISPRi repression strain with gRNA-*suf1*	This study
DJL131	WWM73 att::pDL367	CRISPRi repression strain with gRNA-*suf2*	This study
DJL133	WWM73 att::pDL371	CRISPRi repression strain with gRNA-*suf1* and gRNA-*suf2*	This study
DJL143	WWM73 ∆*sufBC1*	*sufBC1* deletion strain	This study
DJL63	WWM73 ∆*sufBC2*	*sufBC2* deletion strain	This study
DJL142	WWM73 ∆*sufBC2* ∆*sufBC1*	*sufBC1/sufBC2* deletion strain	This study

### CRISPRi repression of *sufC1B1* and/or *sufC2B2* does not impact the growth of *M. acetivorans* with different sulfur and nitrogen sources

**Figure 2:**
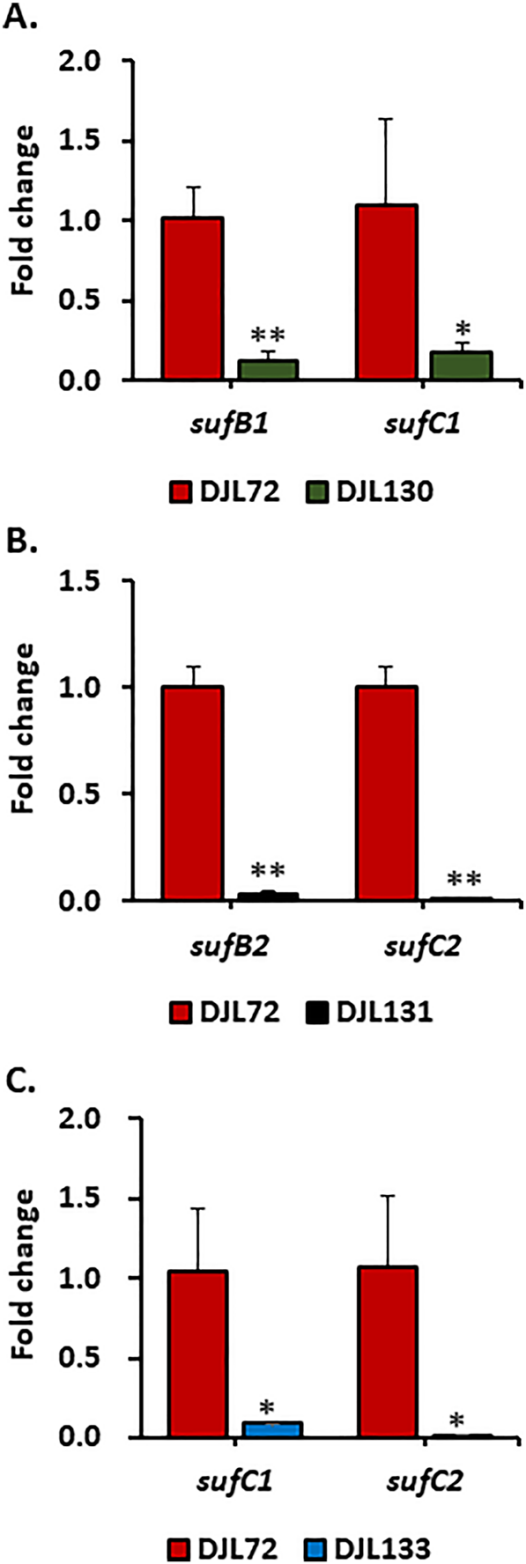
Relative transcript abundance of (**A**) *sufB1* and *sufC1* in DJL130 (**B**) *sufB2* and *sufC2* in DJL131, and (**C**) *sufC1* and *sufC2* in DJL133 as determined by qPCR and compared to strain DJL72 lacking a gRNA (normalized to one). Strains were grown in HS medium containing 125 mM methanol and 1 mM Na_2_S. Error bars indicate mean ± SD for three biological replicates. *p < 0.05, **p < 0.01.

To initially assess the in vivo importance of SufBC in *M. acetivorans*, *sufC1B1* and *sufC2B2* were separately and simultaneously targeted for CRISPRi repression. Separate gRNAs were designed to target the coding strand near the start codon of *sufC1* and the start codon of *sufC2* (Fig. S3). Separate CRISPRi plasmids containing each gRNA (*gRNA-sufC1* or *gRNA-sufC2*) or both gRNAs (Tables 2 and 3) were separately integrated into the genome of *M. acetivorans* strain WWM73 to generate repression strains DJL130 (*gRNA-sufC1*), DJL131 (*gRNA-sufC2*) and DJL133 (*gRNA-sufC1/gRNA-sufC2*) (Table 1). Since we hypothesized that SufBC functions in sulfide-specific Fe–S cluster biogenesis^19^, transformants were selected and maintained in medium with methanol as the carbon/energy source and both cysteine and sulfide as available sulfur sources to minimize selective pressure during strain recovery. The repression of the targeted genes was initially analyzed in cells grown with both cysteine and sulfide using qPCR to determine the relative transcript abundance for *sufB1*, *sufC1*, *sufB2*, and *sufC2* in each repression strain. Relative transcript abundance was compared to strain DJL72, which lacks a gRNA. In each strain, a > 90% reduction in transcript abundance was observed for each targeted *suf* gene (Fig. 2).

**Table 2:** Plasmids used in this study.

Plasmid	Description	Reference
pSK2	*M. acetivorans* expression plasmid	^26^
pDL365	pJA6 with strep-his-*sufC2*	This study
pDN203	*M. acetivorans* CRISPR-Cas9 system plasmid	^24^
pDL734	pDN203 with Cas9 replaced by dCas9	^22^
pDL366	pDL734 with gRNA-*suf1* inserted at AscI site	This study
pDL367	pDL734 with gRNA-*suf2* inserted at AscI site	This study
pDL371	pDL367 gRNA-*suf1* inserted at HpaI site	This study
pDL238	pDN203 without a gRNA	This study
pAMG40	Retrofits with pJK027A-derived plasmids to allow replication in *M. acetivorans*	^25^
pDL248	pUC18 with suf*BC2*-editing DNA (homology repair template + gRNA1-*suf2* + gRNA2-*suf2*)	This study
pDL249	CRISPR-Cas9 plasmid (pDL238) with suf*BC2*-editing DNA	This study
pDL250	pDL249 retrofitted with pAMG40 to allow replication in *M. acetivorans*	This study
pDL381	pUC18 with suf*BC1*-editing DNA (homology repair template + gRNA1-*suf1*)	This study
pDL385	CRISPR-Cas9 plasmid (pDL238) with suf*BC1*-editing DNA	This study
pDL386	pDL385 with gRNA2-*suf1* inserted at HpaI site	This study
pDL387	pDL386 retrofitted with pAMG40 to allow replication in *M. acetivorans*	This study

**Table 3:** gRNAs used in this study.

gRNA	gRNA sequence (+ PAM)	Purpose	Target gene
gRNA-*suf1*	GCTCAATATCAGGTTTTTTA**AGG**	*sufCB1* repression	*sufC1*
gRNA-*suf2*	CCTTAATCTCATTTCAGATA**CGG**	*sufCB2* repression	*sufC2*
gRNA1-*suf1*	GATGGAAAAAAAATTCTGCG**TGG**	*sufCB1* deletion	*sufC1*
gRNA2-*suf1*	GGATTGTCCACCCGGACAAT**CGG**	*sufCB1* deletion	*sufB1*
gRNA1-*suf2*	TGAACCTCGAAGTTGAAAAA**GGG**	*sufCB2* deletion	*sufC2*
gRNA2-*suf2*	TCACCCACGAAGCTGCCGTA**GGG**	*sufCB2* deletion	*sufB2*

**Figure 3:**
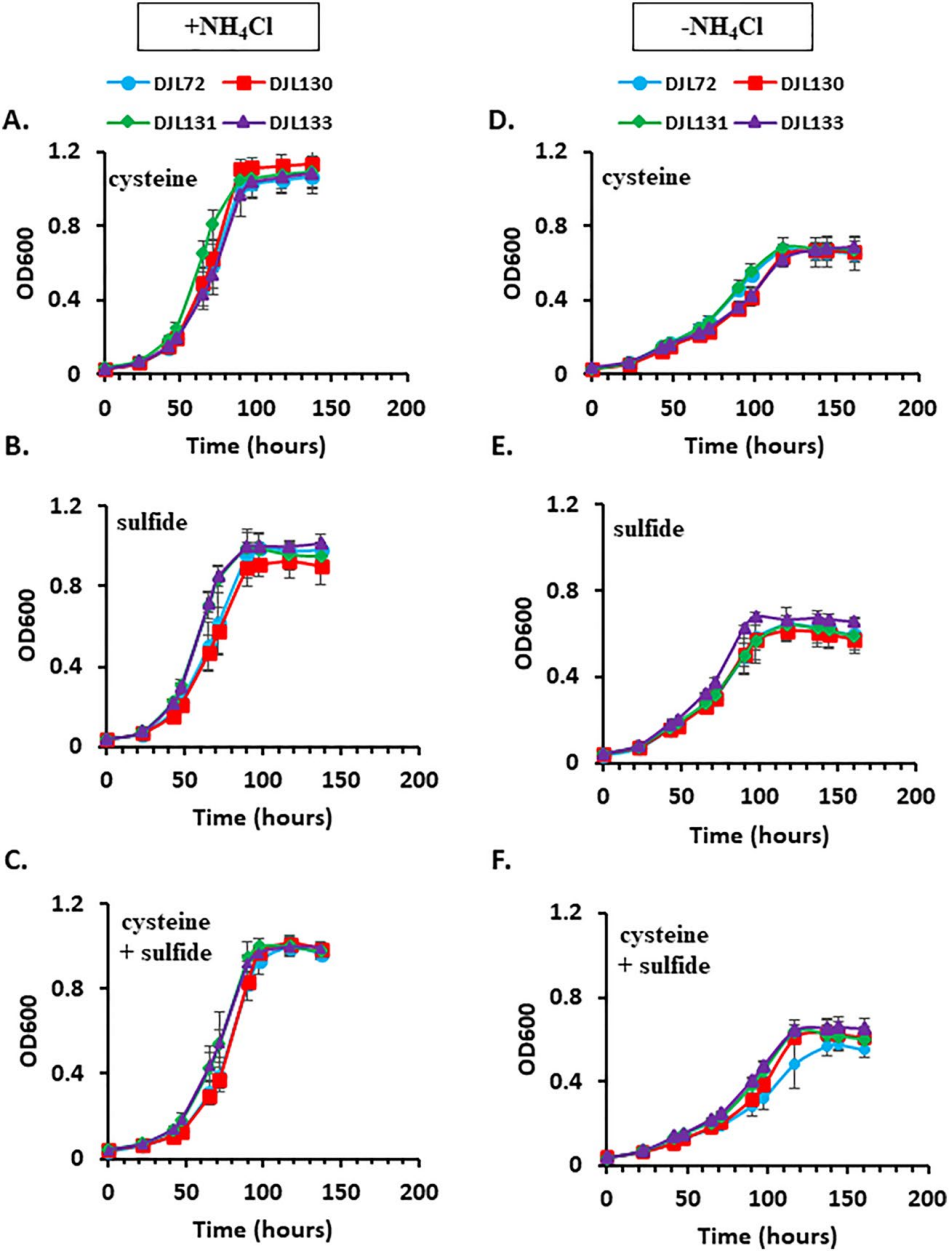
Comparison of the growth of *M. acetivorans* strains DJL130, DJL131, and DJL133 to control strain DJL72 in HS_DTT_ medium containing 125 mM methanol supplemented with cysteine (**A/D**), Na_2_S (**B/E**) or cysteine + Na_2_S (**C/F**) and + NH_4_Cl (left panel) or −NH_4_Cl (right panel). Error bars indicate mean ± SD for at least three biological replicates.

To test the hypothesis that SufBC is required for sulfide-dependent Fe–S cluster biogenesis, the growth of strains DJL130, DJL131, and DJL133 was compared to strain DJL72 (control) with cysteine, sulfide, and cysteine + sulfide as sulfur sources and methanol as the carbon and energy source (Fig. 3A–C). The growth of each CRISPRi repression strain was similar to control strain DJL72, indicating SufC1B1 and SufC2B2 are not involved in sulfur-specific Fe–S cluster biogenesis in *M. acetivorans*. To test the importance of SufC1B1 and SufC2B2 to the assembly of Fe–S clusters in Mo-nitrogenase required for diazotrophy, the growth of the repression strains in medium lacking a fixed nitrogen source (i.e., NH_4_Cl) was compared to control strain DJL72 (Fig. 3D–F). No significant difference was observed during diazotrophic growth of the CRISPRi repression strains compared to the control, indicating SufC1B1 and SufC2B2 are not required for the biogenesis of Fe–S clusters in Mo-nitrogenase. Overall, the ability to significantly repress expression of both *sufC2B2* and *sufC1B1* without any observable phenotype indicates SUF is not the primary Fe–S cluster biogenesis system in *M. acetivorans*.

### SufBC is not essential to *M. acetivorans*

**Figure 4:**
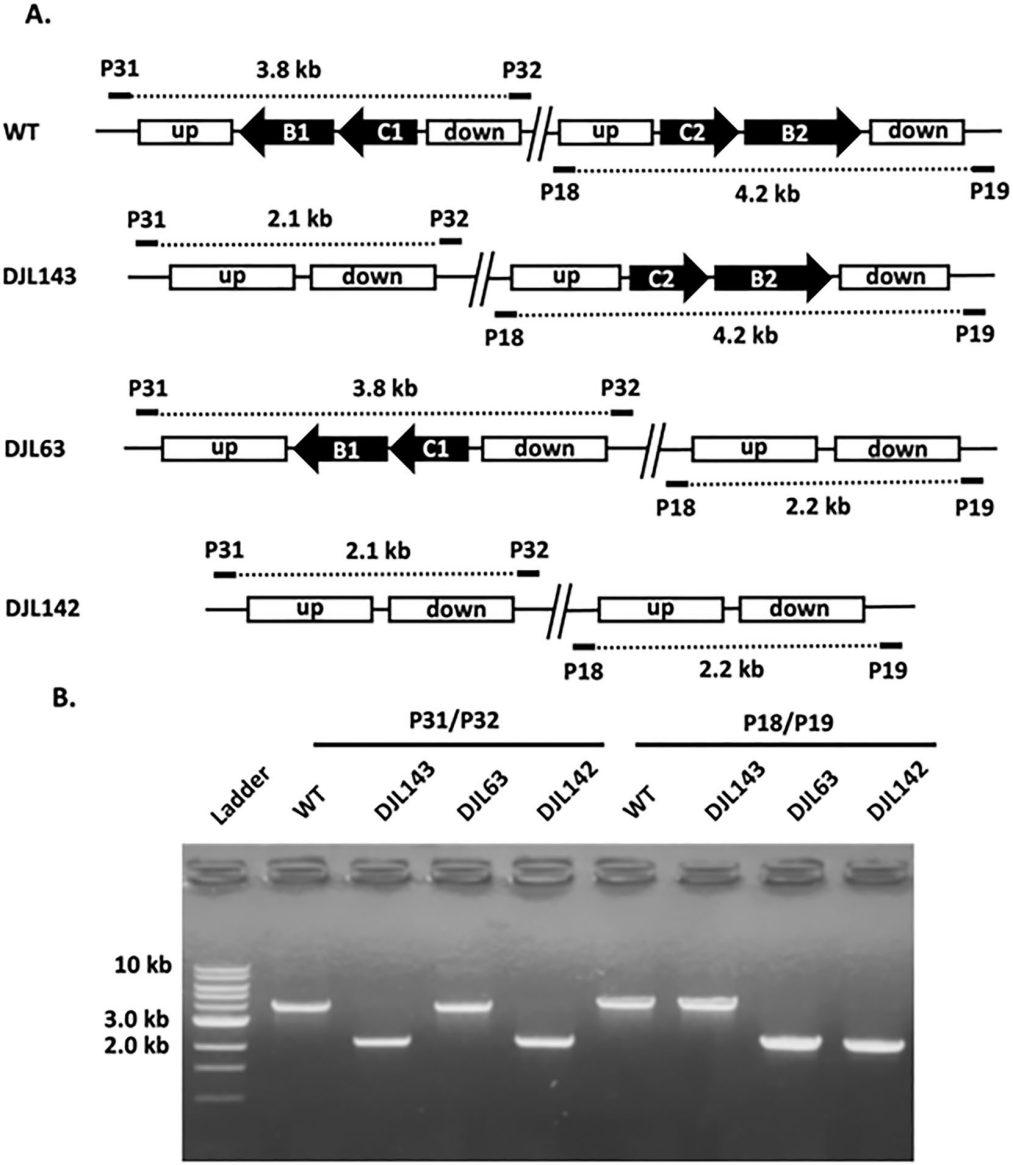
PCR analysis *sufCB1* and *sufCB2* deletion in *M. acetivorans* strains DJL143, DJL63, and DJL142 using primer sets outside the upstream (up) and downstream (down) homology regions. (**A**) Schematic representation showing the size of predicted PCR products with the indicated primers (P#). (**B**) Gel image of PCR products.

**Figure 5:**
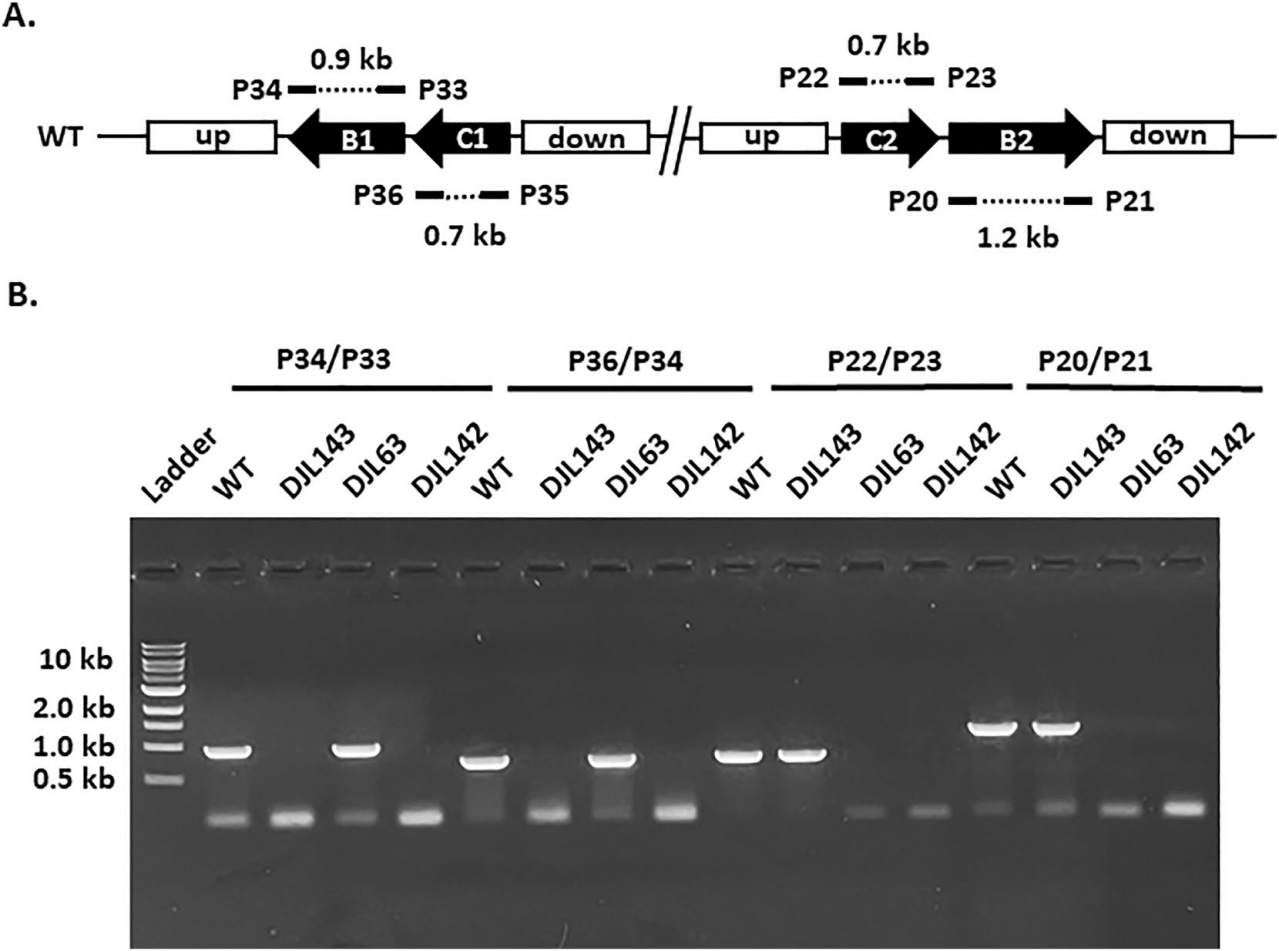
PCR analysis of the deletion of *sufB1*, *sufC1*, *sufB2*, and *sufC2* in *M. acetivorans* strains DJL143, DJL63, and DJL142 using gene-specific primers. (**A**) Schematic representation showing the size of predicted PCR products with the indicated primers (P#). (**B**) Gel image of PCR products.

Despite SufBC being universally conserved in archaea and possibly essential to *M. maripaludis*^17,20^, the dual repression of *sufC1B1* and *sufC2B2* was not only tolerated but did not result in an observable growth phenotype, indicating SufBC does not serve as the primary Fe–S cluster scaffold in *M. acetivorans*. However, it is possible that reduced levels of SufBC are sufficient to support Fe–S cluster biogenesis. Therefore, to unequivocally ascertain the importance of SufBC to *M. acetivorans*, the CRISPR-Cas9 system was used to generate mutant strains deleted of *sufC1B1, sufC2B2* and both *sufC1B1/sufC2B2* (strains DJL143, DJL63, and DJL142, respectively) (Table 1). Genome-edited deletion at the targeted regions in each mutant strain was confirmed using PCR with primers outside of the regions used for homology dependent repair (Fig. 4). The PCR products were also sequenced to confirm precise editing of the region in each deletion mutant, and no unintended mutations were observed. Finally, to confirm complete loss of *sufC1B1* and/or *sufC2B2* from the mutant strains, PCR was performed using gene-specific primers and the corresponding deleted genes were not detected in the appropriate strains (Fig. 5). Importantly, strain DJL142 was obtained that lacks both *sufC1B1* and *sufC2B2*, revealing that the SufBC genes are not essential to *M. acetivorans*.

### SufBC is not required for Fe–S cluster biogenesis in *M. acetivorans*

**Figure 6:**
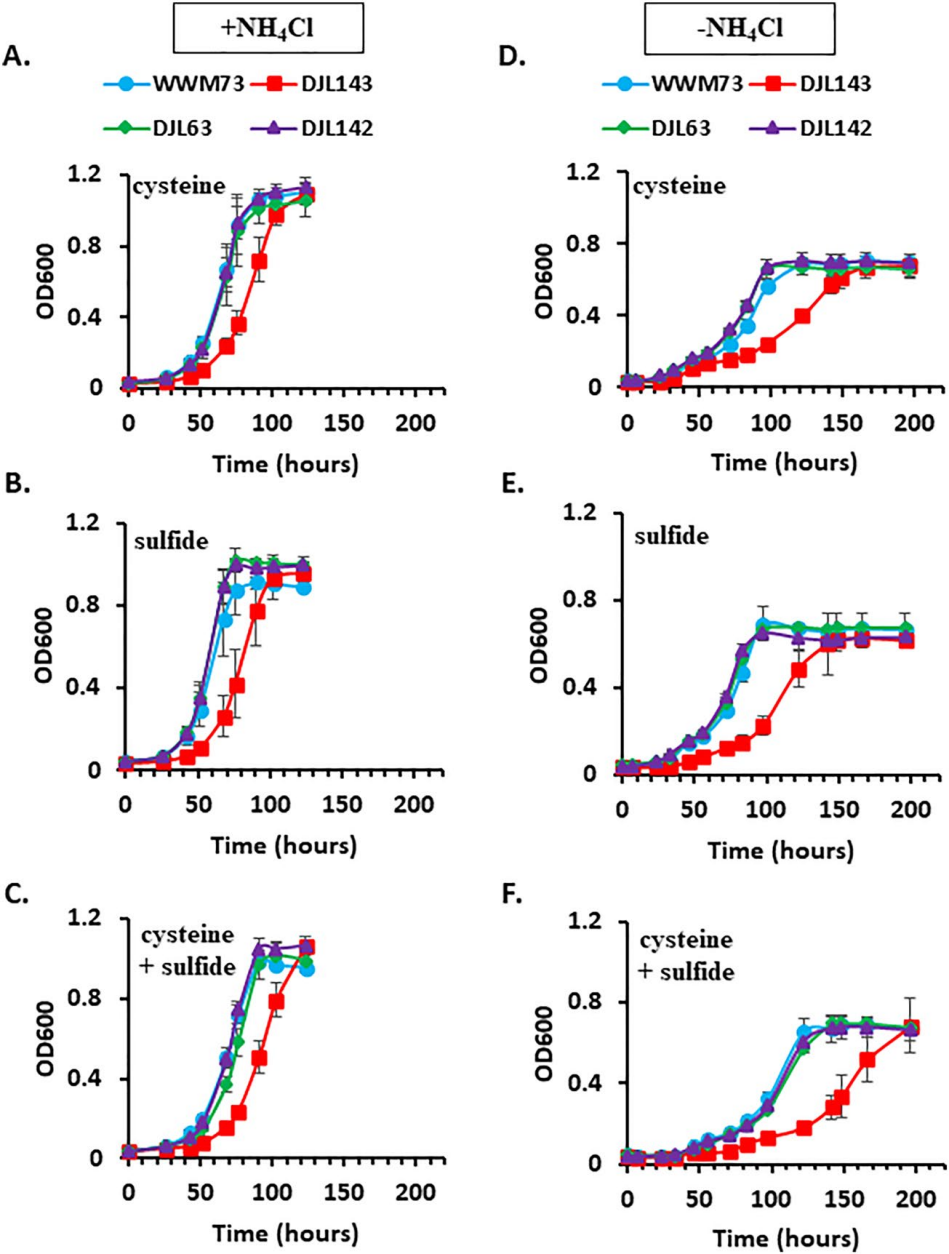
Comparison of the growth of *M. acetivorans* strains DJL143, DJL63, and DJL142 to the parent strain WWM73 in HS_DTT_ medium containing 125 mM methanol supplemented with cysteine (**A/D**), Na_2_S (**B/E**) or cysteine + Na_2_S (**C/F**) and +NH_4_Cl (left panel) or −NH_4_Cl (right panel). Error bars indicate mean ± SD for at least three biological replicates.

**Figure 7:**
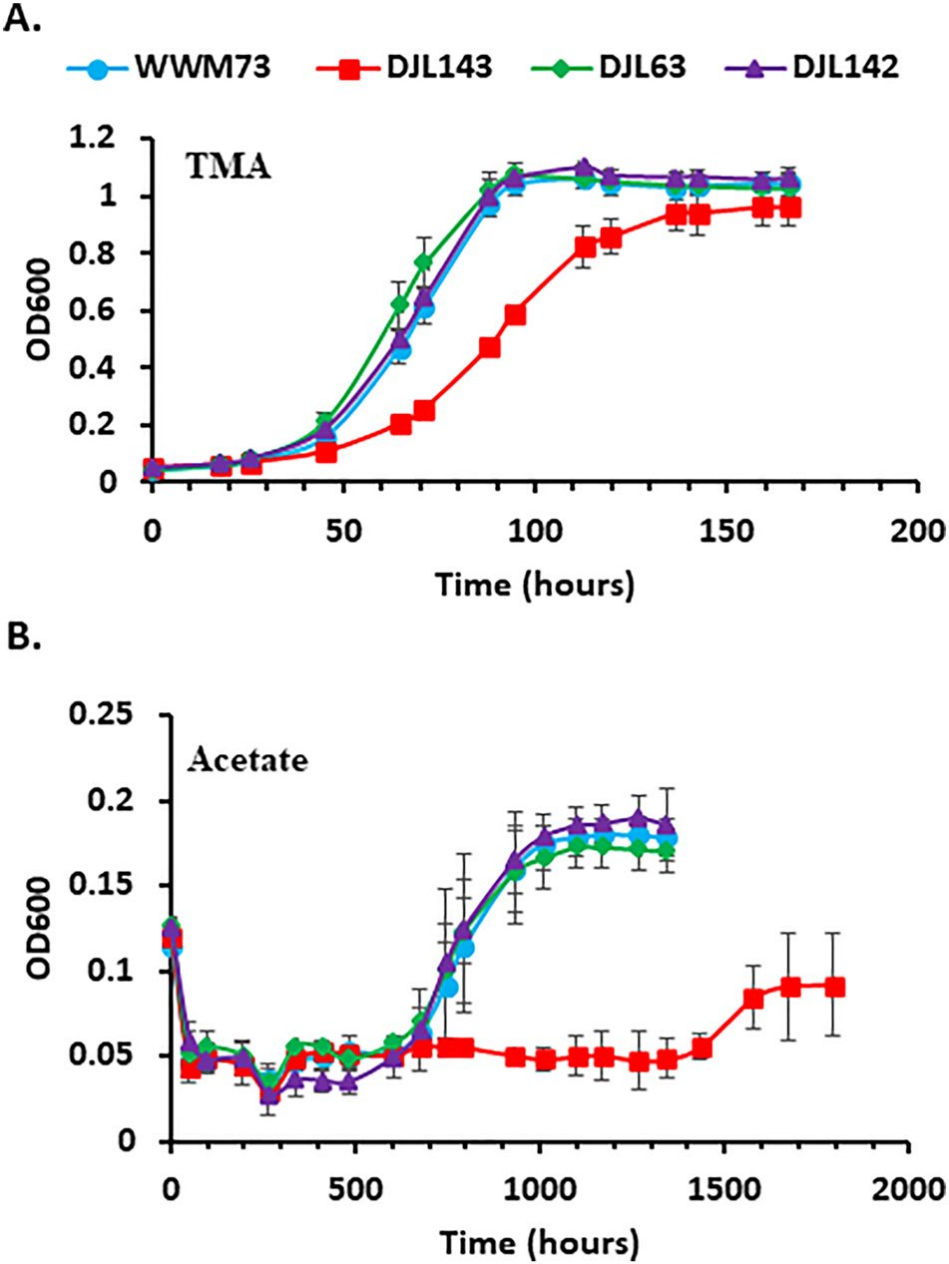
Comparison of the growth of *M. acetivorans* strains DJL143, DJL63, and DJL142 to the parent strain WWM73 in HS_DTT_ medium containing 1 mM Na_2_S, and 18 mM NH_4_Cl supplemented with (**A**) 50 mM TMA and (**B**) 100 mM acetate. Error bars indicate mean ± SD for at least three biological replicates.

To determine the effect of the loss of *sufC1B1* or/and *sufC2B2*, growth of the mutant strains with different carbon, sulfur, and nitrogen sources was compared to *M. acetivorans* strain WWM73 (parent strain). The strains were grown with cysteine and/or sulfide and with NH_4_Cl or N_2_. Methanol was used as the carbon source. As shown in Fig. 6, the growth of strains DJL63 and DJL142 is identical to the parent strain, regardless of the sulfur and nitrogen sources provided. However, strain DJL143 lacking only *sufC1B1* showed a delayed-growth phenotype under all conditions compared to the other strains. The impact of the loss of SufBC on methylotrophic methanogenesis with trimethylamine (TMA) was also tested. The growth of each strain with TMA was similar to that observed with methanol, with strain DJL143 again having a delayed-growth phenotype (Fig. 7A). Finally, the impact of the loss of SufBC on acetoclastic methanogenesis was tested by adapting the mutants to growth with acetate as the carbon and energy source. Cells of the mutant and control (WWM73) strains were transitioned from methanol to acetate as the sole carbon and energy source, by transferring methanol-grown cells to medium containing only acetate. All the strains had an extended adaptation period before the onset of growth (Fig. 7B). However, strain DJL143 again had a much longer delay before the onset of growth compared to the other strains, taking ~ 30 days longer for the onset of growth compared to the other strains. No observable phenotypic difference was observed for strains DJL63 and DJL142 compared to parent strain WWM73.

**Figure 8:**
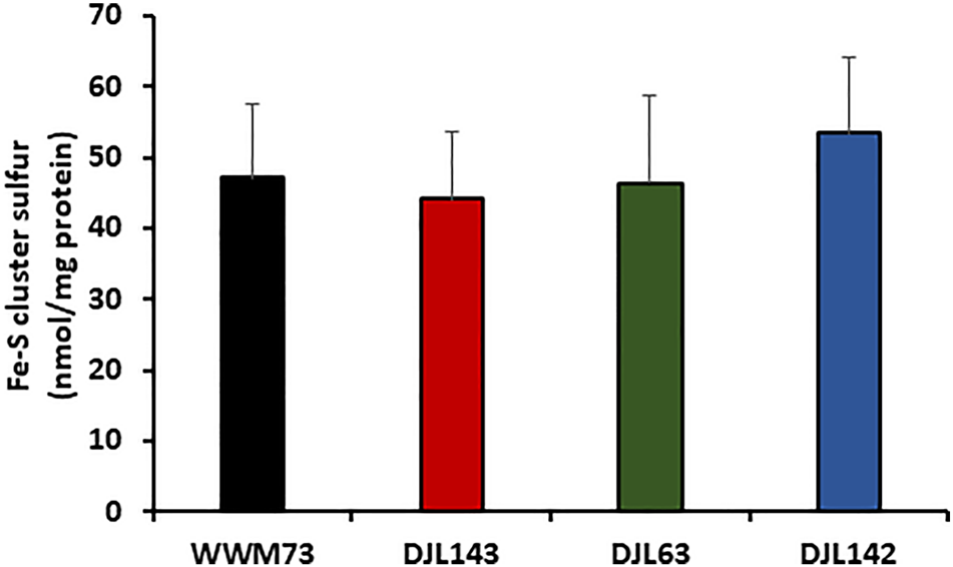
Fe–S cluster content in cell lysates of *M. acetivorans* strains WWM73, DJL143, DJL63, and DJL142 as determined by acid-labile sulfide analysis. Cells were grown in HS_DTT_ medium containing 125 mM methanol, 1 mM Na_2_S, and 18 mM NH_4_Cl. Error bars indicate mean ± SD for three biological replicates.

The ability to completely delete SufBC (strain DJL142) from *M. acetivorans* without producing any observable growth phenotype indicates that the minimal SUF system is not the primary Fe–S cluster biogenesis system, nor is it a sulfide-specific or a nitrogenase-specific Fe–S cluster biogenesis system. Interestingly, strain DJL143 that is only deleted of *sufC1B1* exhibits a consistent delayed growth phenotype. To determine if the phenotype of strain DJL143 is a result of altered Fe–S biogenesis and if the complete loss of SufBC impacts Fe–S protein content, the bulk Fe–S cluster content in cells of strains WWM73, DJL143, DJL63, and DJL142 grown with methanol, sulfide, and NH_4_Cl was determined by measuring the acid-labile sulfur content in cell lysate (Fig. 8). No significant difference was observed in the Fe–S cluster content between the strains, indicating that the observed phenotype of strain DJL143 is not due to altered total Fe–S cluster biogenesis capacity and that SufBC is not a critical component of Fe–S cluster biogenesis overall.

## Discussion

Methanogens are an ancient lineage of anaerobes that arose early on Earth when the atmosphere was likely devoid of oxygen. The absence of oxygen allowed reduced Fe and sulfur to associate to form Fe–S complexes (e.g., pyrite) that drove early energy-conserving reactions^17^. As such, the metabolism of methanogens became highly dependent on Fe–S proteins and continues in modern lineages. Indeed, methanogens contain the largest number of Fe–S cluster proteins^9,11^. Recently, methanogens have been shown to use pyrite as a source of Fe/S and may directly uptake pyrite for the synthesis of Fe–S clusters into proteins^27,28^. However, current evidence supports that all organisms use dedicated protein machinery for the biogenesis of Fe–S clusters in proteins. As such, the presence of SufBC and/or IscSU in methanogens suggests they use similar protein factors as those used by bacteria and eukaryotes. Indeed, we have recently shown that *M. acetivorans* contains a functional minimal ISC system composed of IscSU2^19^. Results from this study demonstrate that *M. acetivorans* SufB2C2 form a complex in vivo consistent with previous results with recombinant protein^18^. However, results from genetic studies clearly reveal that SufBC is not required for Fe–S cluster biogenesis in *M. acetivorans*, challenging the universal role of SufBC in methanogens.

In contrast to results with *M. acetivorans*, a transposon mutagenesis screen indicated SufBC is essential to *M. maripaludis*^20^. *M. maripaludis* is a more deeply rooted methanogen. *M. acetivorans* is more metabolically versatile and is postulated to have acquired additional genes from anaerobic bacteria^29^. Thus, it is possible that SufBC is the primary Fe–S cluster biogenesis system in *M. maripaludis* and other deeply rooted methanogens, but in later-evolving *M. acetivorans* and Methanosarcinales in general, it may serve an auxiliary role. Similarly, the SUF system is essential in some bacteria, such as *Staphylococcus aureus* and *Mycobacterium tuberculosis*, that only contain the SUF system for Fe–S cluster biogenesis^6,30^. In contrast, *E. coli* contains ISC and SUF and the deletion of the *suf* operon is not lethal under normal growth conditions. However, simultaneous deletion of SUF and ISC is lethal in wild type *E. coli*. Moreover, the SUF system has the ability to complement for the loss of the ISC system^31^. *M. acetivorans* also contains a minimal ISC system, hence, it is possible that SUF is not essential in organisms that contain additional Fe–S cluster biogenesis systems.

The acquisition of the minimal ISC system by *M. acetivorans* likely expanded the ability to use cysteine, as well as sulfide and pyrite as a direct source of sulfur for Fe–S cluster biogenesis. Indeed, loss of IscS2U2 in *M. acetivorans* confers a cysteine-specific growth defect^19^. In contrast, *M. maripaludis* can only use sulfide as the sulfur donor for Fe–S cluster biogenesis and cannot use cysteine^11^. The results of these studies led us to hypothesize that IscSU is the primary Fe–S cluster biogenesis system when cysteine is the sulfur source while SufBC might be the major player during growth with sulfide. However, the results presented here do not support the hypothesis, since a sulfide-specific phenotype was not observed with any of the *M. acetivorans suf* mutants. Interestingly, deletion of *sufC1B1* resulted in a delayed growth phenotype under all the conditions tested, and the phenotype is restored back to wild type when both *sufC1B1* and *sufC2B2* are deleted, indicating deletion of *sufC2B2* acts as a suppressor mutation. However, given the lack of a significant alteration of the bulk Fe–S cluster content in any of the *sufBC* deletion mutants (Fig. 8), the delayed growth phenotype appears unlikely to be directly related to the Fe–S cluster biogenesis. It is possible that SufBC does not function in Fe–S cluster biogenesis in *M. acetivorans* or methanogens in general, despite the in vitro evidence with recombinant SufB2C2^18^. Additional experimentation is required to determine the specific in vivo function of SufBC in methanogens.

Nitrogenase is composed of two components, dinitrogenase reductase (NifH) and dinitrogenase (NifDK). NifH is a homodimer with a single [4Fe–4S] cluster required for electron transfer to the [8Fe–7S] cluster (P-cluster) in NifDK. Electrons are then transferred to the [7Fe-9S-Mo-C-homocitrate] cluster called the M-cluster or FeMo-co in NifDK. FeMo-co is the site of N_2_ reduction to ammonia. Importantly, [4Fe–4S] clusters are not only needed for NifH but also for the biogenesis of the P-cluster and FeMo-co^32,33^. In bacteria, such as the model diazotroph *Azotobacter vinelandii*, NifSU are required for providing these [4Fe–4S] clusters^34^. SufBC has not been shown to function in the biogenesis of [4Fe–4S] clusters needed for nitrogenase maturation. Nonetheless, *M. maripaludis* contains Mo-nitrogenase, and diazotrophy is well characterized in this methanogen^35–37^. Since *M. maripaludis* lacks NifSU and IscSU, it must use SufBC or an unknown factor for the biogenesis of [4Fe–4S] clusters needed for nitrogenase maturation. However, results here indicate that SufBC is not involved in Mo-nitrogenase Fe–S cluster biogenesis in *M. acetivorans*. This raises the important question of whether maturation of nitrogenase in *M. acetivorans* involves the IscSU system or an unknown Fe–S cluster biosynthesis system. However, the *M. acetivorans iscS2U2* deletion mutant exhibits identical diazotrophic growth compared to wild-type *M. acetivorans* when provided sulfide and only impaired growth with cysteine (Tom Deere, personal communication), indicating other factors may be involved.

While SufBC is universally conserved in archaea, the results presented here do not support its role in Fe–S cluster biogenesis in *M. acetivorans*. Overall, the results presented in this study lead to the following possibilities—(1) the Fe–S cluster biogenesis function of SufBC is redundant in *M. acetivorans*, or (2) SufBC is not involved in Fe–S cluster biosynthesis and serves a different unknown function in this methanogen. Considering SufBC are likely ancient Fe–S cluster biogenesis components present in the last universal common ancestor (LUCA)^18^, it is possible that the appearance of other Fe–S cluster biosynthesis machineries such as ISC and NIF in bacteria and archaea resulted in the redundancy of SUF. For example, *Candidatus Methanoplasma termitum* is an example of a methanogen that lacks SufBC^38^. Clearly, considerable knowledge gaps remain regarding the mechanisms of Fe–S cluster biogenesis in methanogens, and it is highly likely that additional factors are involved that await discovery in these ancient and biotechnologically important anaerobes.

## Materials and methods

### *M. acetivorans* strains and growth

*M. acetivorans* strain WWM73 was the parent strain for all experiments. All strains of *M. acetivorans* are listed in Table 1. The strains were grown in anoxic high-salt (HS) medium at 35º C as previously described^39^. HS medium was prepared inside an anaerobic chamber (Coy Laboratories) containing 75% N_2_, 20% CO_2_, and 5% H_2_. The medium was supplemented with 2 µg/mL puromycin when required. Growth experiments were performed in Balch tubes containing 10 ml of HS medium reduced with 1.5 mM DTT. 125 mM methanol or 50 mM TMA or 100 mM acetate, 1 mM sulfide and/or 3 mM cysteine and 18 mM NH_4_Cl were added from anaerobic sterile stock solutions prior to inoculation where indicated. The optical density was measured at 600 nm using a spectrophotometer.

### Construction of *M. acetivorans sufCB* CRISPRi repression strains

The CRISPRi system was used to repress *sufC1B1* (*ma0937-ma0936*) and/or sufC2B2 (*ma4406-ma4407*) in *M. acetivorans* parent strain WWM73. All primers (Table S1) and gBlocks were designed using Geneious Prime software and purchased from Integrated DNA Technologies (IDT). For the construction of CRISPRi-dCas9 plasmids with a single gRNA, a gBlock containing either gRNA-*suf1* or gRNA-*suf2* (Table 3) was introduced into pDL734 separately as previously described^22^. Briefly, pDL366 and pDL367 (Table 2) were constructed by digesting pDL734 with *Asc*I (New England Biolabs). The gBlock was then introduced into digested pDL734 using a Gibson Assembly Ultra Master mix kit (Codex DNA) as per the manufacturer’s instructions. *E. coli* WM4489 competent cells were transformed with Gibson Assembly reactions. Transformants were screened by PCR using primers P1 and P2. Next, *M. acetivorans* WWM73 was separately transformed with pDL366 and pDL367 using liposome-mediated transformation method^40^. Transformants were selected on anaerobic HS agar plates containing 125 mM methanol and 2 µg/mL puromycin. The plates were incubated at 35º C in an anoxic mason jar containing 2.5 mL of 2.5% w/v sulfide in a vial and incubated. Colonies were screened by PCR using two sets of primers P3/P4 and P5/P6 and positive colonies were selected and designated as DJL130 (pDL366) and DJL131 (pDL367). The strains were maintained in HS medium containing 125 mM methanol, 1 mM sulfide and 2 µg/mL puromycin.

For the construction of CRISPRi-dCas9 plasmid with two gRNAs (pDL371), pDL367 was digested with HpaI (NEB) and a gBlock containing gRNA-*suf1* was introduced into *Hpa*I-digested pDL367 using Gibson Assembly. Transformants were screened by PCR using primers P7 and P8. Next, *M. acetivorans* WWM73 was transformed with pDL371 as described above. Colonies were screened by PCR using two sets of primers P3/P4 and P5/P6 and positive colony was selected and designated as DJL133. The strain was maintained in HS medium containing 125 mM methanol, 1 mM sulfide and 2 µg/mL puromycin.

### Gene expression analysis

*M. acetivorans* cells were harvested at mid-log phase (OD_600_ of 0.4–0.5) by anaerobic centrifugation. Pellets were resuspended in 1 mL Trizol reagent (Ambion, Life Technologies) and stored at -80º C. RNA was extracted using the Direct-zol RNA MiniPrep kit (Zymo Research) followed by DNase treatment using the DNA-free DNA Removal kit (Invitrogen, Thermo Fisher Scientific). cDNA was synthesized from 300 ng of RNA using the iScript Select cDNA Synthesis kit (Bio-Rad). Gene expression analysis was done by qPCR using cDNA (300-fold dilution) and SsoAdvanced Universal SYBR Green Supermix (Bio-Rad). Primers used for qPCR were designed using Geneious Prime software and purchased from IDT. *sufB1* expression was analyzed using primers P41/42, *sufC1* expression using primers P43/44, *sufB2* expression using primers P45/46, and *sufC2* expression using primers P47/48. Primers P39 and P40 were used to measure the expression of 16 s rRNA (an internal control). The reactions were carried out in a CFX96 Real-Time PCR Detection system (Bio-Rad). The data were analyzed using ∆∆Cq calculation method.

### Generation of *M. acetivorans sufBC* deletion mutant strains

All deletion strains of *M. acetivorans* were generated using a CRISPR-Cas9 system with a few modifications^24^. All primers and gBlocks were designed using Geneious Prime software and purchased from IDT. Strain DJL63 was generated by introducing *sufC2B2*-editing DNA (homology repair template + gRNA1-*suf2* + gRNA2-*suf2*) into pUC18 followed by introducing it to pDL238. Briefly, homology regions upstream and downstream of *sufC2B2* were amplified by PCR using primers P9/10 and P11/12 respectively. Plasmid pDL248 was constructed by digesting pUC18 with *Bam*HI-HF and introducing suf*C2B2*-editing DNA into digested pUC18 using Gibson Assembly. *E. coli* DH5α competent cells were transformed with Gibson Assembly reaction. Transformants were screened by PCR using P9 and P13. The suf*C2B2*-editing DNA was amplified by PCR using Q5 polymerase, primers P14/P15 and pDL248 as template. The amplified suf*C2B2*-editing DNA was introduced into *Asc*I-digested pDL238 using Gibson Assembly followed by transformation into WM4489 competent cells. Transformants were screened by PCR to confirm the presence of plasmid pDL249 using primers P9 and P13. pDL249 was retrofitted with pAMG40 using Gateway BP Clonase II enzyme mix (Invitrogen) followed by transformation into WM4489 cells. Transformants were screened by PCR using the primer set P16/P17 and the plasmid was designated pDL250. Next, *M. acetivorans* WWM73 cells were transformed with pDL250. Transformants were selected on anaerobic HS agar plates containing 125 mM methanol and 2 µg/mL puromycin as described above. Colonies were screened by PCR using primers that anneal outside the homology region (P18 and P19) and gene-specific primers (P20/21 for *sufB2* and P22/23 for *sufC2*). To construct a markerless deletion mutant, cells were plated on HS plates containing 125 mM methanol and 50 µg/mL 8-Aza-2,6-diaminopurine sulfate (8-ADP) as described^24^. The colonies cured of the plasmid were screened by PCR using primers P24 and P25.

For the construction of DJL143 (*suf1* deletion mutant) and DJL142 (*suf1/2* deletion mutant), homology regions upstream and downstream of *sufC1B1* were amplified by PCR using primers P26/27 and P28/29 respectively. Plasmid pDL381 was constructed by digesting pUC18 with *Bam*HI-HF and introducing suf*B1C1*-editing DNA (homology repair template + gRNA1-*suf1*) into digested pUC18 using Gibson Assembly. *E. coli* DH5α competent cells were transformed with Gibson Assembly reaction. Transformants were screened by PCR using primers P13 and P26. The *sufC1B1*-editing DNA was amplified by PCR using Q5 polymerase, primers P15/30 and pDL381 as template. The amplified *sufC1B1*-editing DNA was introduced into *Asc*I-digested pDL238 using Gibson Assembly followed by transformation into WM4489 competent cells. Transformants were screened by PCR to confirm the presence of plasmid pDL385 using primers P15 and P26. To introduce the second gBlock containing gRNA2-*suf2*, pDL385 was digested with *Hpa*I and gRNA2-*suf2* was introduced into *Hpa*I-digested pDL385 using Gibson Assembly followed by steps mentioned above to construct a retrofitted plasmid (pDL387). *M. acetivorans* WWM73 and DJL63 were separately transformed with plasmid pDL387 to generate *suf1* (DJl143) and *suf1/2* (DJL142) deletion mutants. Transformants were selected on anaerobic HS agar plates as described above. Colonies were screened by PCR using primers that anneal outside the homology region (P31 and P32) and gene-specific primers (P33/34 for *sufB1* and P35/36 for *sufC1*). To construct a markerless deletion mutant, cells were plated on HS plates containing 125 mM methanol and 50 µg/mL 8-ADP (Biosynth). The colonies cured of the plasmid were screened by PCR using primers P24 and P25.

All plasmids transformed into *M. acetivorans* were verified by DNA sequencing (Plasmidsaurus Sequencing). Additionally, deletions were confirmed by sequencing PCR-amplified upstream and downstream regions around the deleted genes.

### Determination of total Fe–S cluster content in *M. acetivorans* cell lysate

The total Fe- S cluster within *M. acetivorans* strains was determined by measuring total acid-labile sulfur content using the methylene blue method as previously described^41^. *M. acetivorans* cells were harvested at mid-log phase (OD_600_ of 0.4–0.5) by anaerobic centrifugation (8000×*g* for 15 min at 4 °C). The cell pellet was resuspended in buffer containing 50 mM Tris, 150 mM NaCl, 1 mM benzamidine, and 1 mM phenylmethylsulfonyl fluoride and stored at -80º C using anaerobic vials. Cell pellets were lysed anaerobically by sonication on ice using a sonicator (QSonica) followed by anaerobic centrifugation (14,000×*g* for 10 min). The cell lysate was collected, and the total protein concentration was determined using Qubit protein assay kit (Molecular probes, Life Technologies). The assay was performed in sealed vials containing protein samples. 1% zinc acetate and 12% sodium hydroxide were added to the vials containing samples and incubated at room temperature for 2 h. The reactions were terminated by the addition of 5 mM N,N-Dimethyl-p-phenylenediamine dihydrochloride in 5 M HCl and 0.23 M FeCl_3_ in 1.2 N HCl followed by incubation at room temperature for 2 h. The vials were quickly vented, solutions were spun down at 16,000 × g and absorbances were read at 670 nm using a spectrophotometer.

### Construction of a strep-SufC2B2 expression strain of *M. acetivorans*

PCR was used to amplify *sufC2B2* from *M. acetivorans* genomic DNA using Q5 high-fidelity DNA polymerase (NEB) as per the manufacturer’s instructions. Primers were designed and purchased from IDT. Restriction site *Nco*I was added at the 5’ end of *sufC2* and restriction site *Nru*I at the 3’ end of *sufB2* using primers P37 and P38. The PCR product and plasmid pSK2^26^ were digested with *Nco*I and *Nru*I using standard digestion protocol (NEB). Digested PCR product and pSK2 were gel purified as per manufacturer’s instructions (Qiagen) followed by ligationusing T4 DNA ligase. *E. coli* DH5α competent cells (NEB) were transformed with the ligation reaction. Transformants were screened by PCR using primer set P37/38 and confirmed by DNA sequencing (Eurofins). The pSK2 plasmid containing the *sufC2B2* gene sequence was designated as pDL365. *M. acetivorans* strain WWM73 was transformed with pDL365 and pSK2 (control plasmid that expresses strep-UidA) using a liposome-mediated transformation protocol as previously described^40^. Transformants were selected on HS agar plates as described above. Transformants were screened by PCR for the presence of strep-*sufC2B2*, and a positive transformant designated as strain DJL66 and maintained in HS medium containing 125 mM methanol, 1 mM sulfide and 2 µg/mL puromycin.

### Western blot analysis

Western blot analysis was used to detect strep-tagged protein in strain DJL66 expressing strep-SufC2 and in control strain WWM73 (pSK2) expressing strep-UidA. *M. acetivorans* cells were harvested at mid-log phase (OD_600_ of 0.4–0.5) by aerobic centrifugation (8000×*g* for 10 min at 4 °C). The cell pellet was resuspended in buffer A containing 50 mM Tris, 150 mM NaCl, 1% Tween, 1 mM Benzamidine, 2 mM EDTA and sonicated 3 times on ice using a sonicator (QSonica) followed by aerobic centrifugation (8000×*g* for 10 min at 4 °C). The cell lysate was collected, and the total protein concentration was determined using Bio-Rad protein assay^42^. Protein sample (15 µg) was resolved in a 12% SDS-PAGE gel and transferred to a nitrocellulose blotting membrane (Amersham Protran 0.1 µm NC; GE Healthcare Life Sciences). The membrane was blocked for 20 min in blocking buffer containing 50 mM Tris, 150 mM NaCl, 0.1% Tween (TBST), 5% milk and incubated overnight with the α-strep antibody (Qiagen). The membrane was washed three times with goat anti-mouse IgG antibody (GenScript) for one hour followed by washing three times with TBST again. The membrane was developed with Western blotting substrate (Thermo Scientific) and scanned using a FlourChem 8900 imaging system (Alpha Innotech).

### Purification and characterization of strep-SufC2

*M. acetivorans* cells were harvested at an OD_600_ of 0.8 by anaerobic centrifugation (8000×*g* for 10 min at 4 °C) and the pellets were stored at -80ºC. For purification of recombinant protein, all the steps were carried out in an anaerobic chamber (Coy Laboratories) containing 95% N_2_ and 5% H_2_. The cells were thawed and resuspended in buffer NP (50 mM NaH_2_PO_4_ and 300 mM NaCl, pH 8.0) containing 4 μg/mL DNase I and 1 mM Benzamidine. The cells were sonicated 3 times followed by centrifugation (8000×*g* for 10 min at 4 °C). The cell lysate was loaded on a chromatography column containing 1 mL of Strep-Tactin superflow plus resin (Qiagen) pre-equilibrated with 12 mL of buffer NP. The column was washed with 5 ml buffer NP. Protein was eluted in 3 mL buffer NP containing 2.5 mM desthiobiotin (NPD) and stored at − 80 °C. The protein concentration was determined using the Bio-Rad protein assay^42^. The purified protein was analyzed by 12% SDS-PAGE gel stained with Coomassie blue solution followed by de-staining. The protein bands were excised, trypsin digested, and sent for mass spectrometry (The University of Arkansas Statewide Mass Spectrometry Facility). UV–visible absorption spectrum was recorded using a Cary 60 spectrophotometer (Agilent Technologies) under anaerobic conditions.

### Supplementary Information

Supplementary Information.

## Data Availability

The raw data from growth studies and qPCR is available upon request.
