# Supplementary material for: The minimal SUF system is not required for Fe–S cluster biogenesis in the methanogenic archaeon Methanosarcina acetivorans
Source: Sci Rep. 2023 Sep 13;13:15120. doi: 10.1038/s41598-023-42400-x (PMC10500019; doi:10.1038/s41598-023-42400-x)
Supplement: Supplementary file 1 — Supplementary Information. [file 41598_2023_42400_MOESM1_ESM.pdf]

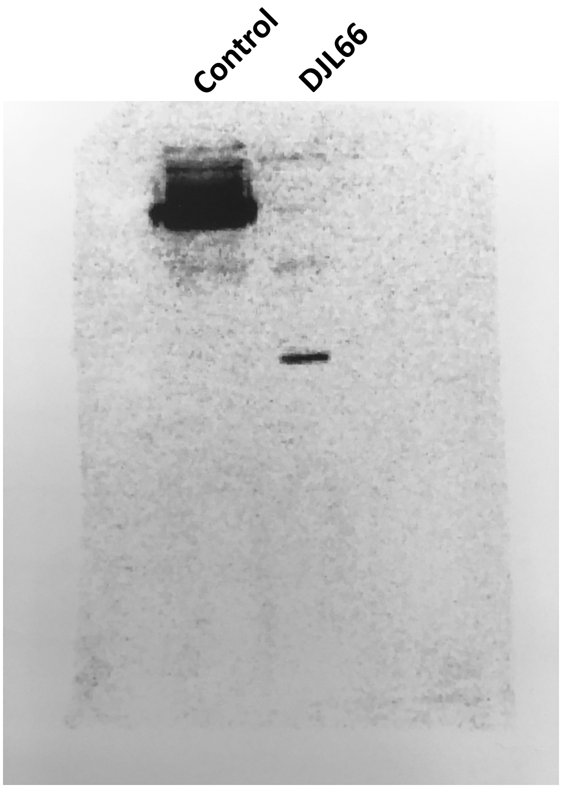

Figure S1. Uncropped image of Western blot shown in Fig. 1A.

A.

MLKIEDLTVEVDGK**TLLHDVNLEVEKGYTNVLFGPNGAGK**SALMR**TIMGF**
SEYRVVKGRILFKGEDITHLPIDERARLGLGIMMQRPPDMAGIKLKDLVK
VASKGKKDPETLAENLDMKR**FLDRDVNVGFSGGEIK**RSELLQLSAQNPSL
YLLDEPESGVDLVSIEQVGMTIKELLEEGLECPGERCKKGK**SALIITHT**G
QVLDYVQADRGYILCNGTVMCSGNPMKMLEEIKNKGYQECITCKLMK

B.

MQTDEMSLKKRAESAAEKKAAYGEDFELEKFEEGSKVSKPIEDLQTLDEE
SKKTLLQVGIIPSEEGRSGSFIVLDNAVSHSSLKDENVELMSTHKALEK**Y**
EWLKDYSWKLVQVDADKYTAK**TYLEDADGYFIR**APPGKKSSMPVQTCLML
GSKKAAQTVHNIIVVEEEASLDIITGCTTKKGVEEGLHLGISEMYVKKGA
TLNFTMIHNWAEQIGVRPRTVVHVEEGGTYISNYICLKPVHSVQTYPTVR
LEGKGAVTR**LNTIAIGHPGSELDLGSRAVFNAPDTR**AELISRTITIGGRL
VARGEMIGNAKGAKGHLECKGLVLTDKGSQLAIPILEANVDDIELTHEAA
VGKIAKDQVEYLMAR**GLTEDEAVGMIIRGFLDVGIR**GIPEELKKEIEETI
TQTALGM

Figure S2. Peptides unique to (A) SufC2 and (B) SufB2 shown in red, as identified by mass spectrometry of the excised ~30 kDa protein band and ~50 kDa protein, respectively, from the SDS-PAGE gel in Figure 1.

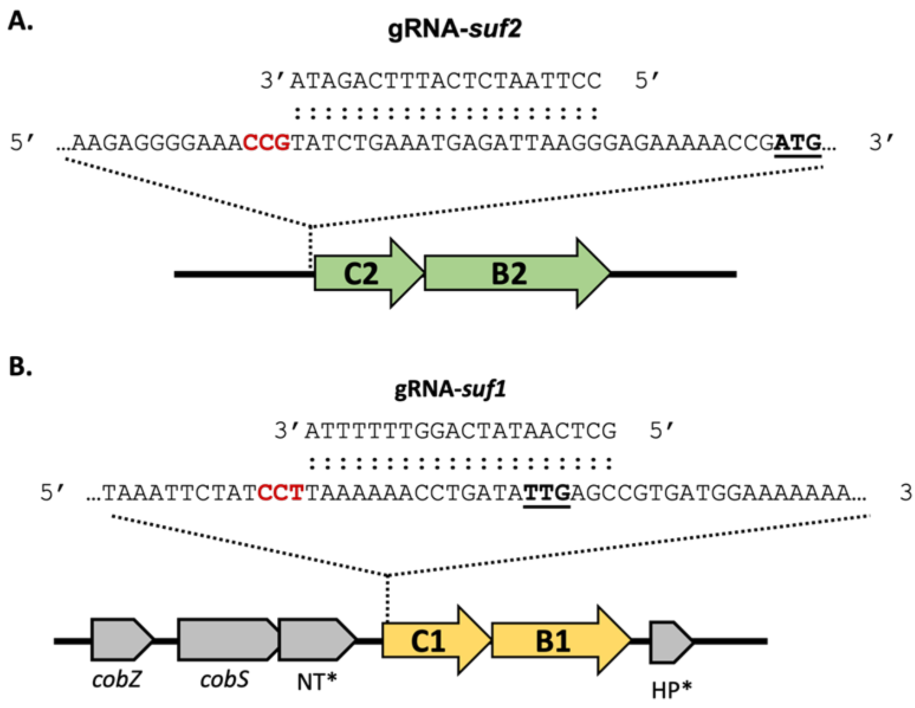

Figure S3. gRNAs designed for CRISPRi-dCas9 repression. (A) gRNA targeting the 5' end of *sufCB2* operon before the start codon. (B) gRNA targeting the 5' end of *sufCB1* operon at the start codon. Neighboring genes are shown in grey. HP* Hypothetical protein; NT* Nucleotidyl transferase.

Table S1. Primers used in this study.

Primer	Sequence (5' to 3')
P1	CAGCTAGCTAACGCGTATTAAAGG
P2	GCCATTTTTCTTCTCACCGGGGAGCTGAGC
P3	GAAGCTTCCCCTTGACCAAT
P4	TTTTTCGTCTCAGCCAATCC
P5	TTGATTCGGATACCCTGAGC
P6	TTGATTCGGATACCCTGAGC
P7	AACGGAGTAACCTCGGTGTGC
P8	AGTTCGGTGCCCAGGAGC
P9	CAAGCTTGCATGCCTGCAGGTCGACTCTAGAGGATCCATATGGAGAAAA TTGGTATC
P10	GACTTACATCGGTTTTTCTCCCTTAATCTCATTTC
P11	GAGAAAAACCGATGTAAGTCCCGGCACC
P12	GACTGATGTTGTTGGCGCGCCTGCAGGT TTAAACCCTGTCCGAGTCTGTCAGCC
P13	CATGATTACGAATTCGAGCTCGGTACCCGGGGATCCGTGAGTCGTATTA ATTAAGCGGCC
P14	GCCTTTTTTTTTCGAAGTTTAAACCTGCAGGCGCGCCATATGGAGAAAAT TGGTATCAAC
P15	GTGAGTCGTATTAATTAAGCGGCCG
P16	GCGCACCGTGGGCTTGTACTCGGTC
P17	TCGCCTTCTTGACGAGTTCTTCTGAGCGGG
P18	GCTCAGACTGACAGTGCTGTACG
P19	CTCCTATCTGTCCAGGGAAATGCC
P20	GGTGGTCATATGCAAACTGATGAAATGAGCCTG
P21	GGTGAATTCTTACATCCCGAGAGCGGTTTGTGT AATCG
P22	GGTGGTCATATGCTGAAAATAGAAGATCTGACTG
P23	GGTGGATCCTCATTTCATCAGTTTGCATGTTATGC
P24	CTGAGTTCTGATTTTAGTTCTAGACC
P25	TCAATCACCCCCAAACATCATG
P26	AAGCTTGCATGCCTGCAGGTCGACTCTAGAGTTCCAAAGTCATGTCTGA GAAC
P27	TGTCATAAATTCTATCCTTAAAAAACCTGATAAACCTGGCATTCTATTCC TTTTATATTC
P28	TATCAGGTTTTTTAAGGATAGAATTTATGACACC
P29	AAATGACAATAAACGGAGCAAC
P30	TTTCGAAGTTTAAACCTGCAGGCGCGCCTTCCAAAG TCATGTCTGAGAAC
P31	TCAAACAACGTGCCTACCTGATTC
P32	TTGGTTCCAATCTTCCGCTTC
P33	GGTGGTCATATGACTCAGATTACATTGAATGC
P34	GGTGGTCTCGAGTTAAGCCAGCATTCCCTTTACTATGATGTC
P35	GGTGGTCATATGAGCCGTGATGGAAAAAAAATTCTGCG

P36	GGTGAATTCTCAGACCACCTTCGGCGGTGATACCCGGC
P37	GGTGGTCCATGGCACTGAAAATAGAAGATCTGACTG TAGAGGTTG
P38	GGTGGTTCGCGATTACATCCCGAGAGCGGTTTGTGTAATCG
P39	GGTACGGGTTGTGAGAGCAA
P40	CTCGGTGTCCCCTTATCACG
P41	CGTTGTCGGTTATGGCAATCC
P42	TGGTCACCAAGGTCTACGGA
P43	GCAATTTCTTCCCTGTGCGTAA
P44	TCCTTGATGAGCCGGATTCC
P45	TGAAGAAGGCTCCAAGGTCAG
P46	AAACTGCCGGATCTGCCTTC
P47	AATGGAGCCGGAAAGTCAGC
P48	CGGGCTCTTTCATCAATCGGA
